# A case report of isolated arrhythmogenic left ventricular cardiomyopathy: phenotypes, diagnosis, and treatment

**DOI:** 10.1093/ehjcr/ytad581

**Published:** 2024-02-07

**Authors:** Yang Lan, Lai Wei, Cuizhen Pan, Tzuchun Lin, Yan Yan

**Affiliations:** Department of Cardiology, Shanghai Institute of Cardiovascular Diseases, Zhongshan Hospital, Fudan University, 180 Fenglin Rd, Shanghai 200032, China; Department of Cardiovascular Surgery, Zhongshan Hospital, Fudan University, Shanghai 200032, China; Department of Echocardiography, Zhongshan Hospital, Fudan University, Shanghai 200032, China; Department of Cardiology, Shanghai Institute of Cardiovascular Diseases, Zhongshan Hospital, Fudan University, 180 Fenglin Rd, Shanghai 200032, China; Department of Cardiology, Shanghai Municipal Hospital of Traditional Chinese Medicine, Shanghai University of Traditional Chinese Medicine, Shanghai 200071, China; Department of Cardiology, Shanghai Institute of Cardiovascular Diseases, Zhongshan Hospital, Fudan University, 180 Fenglin Rd, Shanghai 200032, China

**Keywords:** Case report, Isolated arrhythmogenic ventricular cardiomyopathy, Ventricular tachycardia, Left ventricular aneurysm

## Abstract

**Background:**

Isolated arrhythmogenic left ventricular cardiomyopathy (IALVC) is a hereditary cardiomyopathy that is characterized by the replacement of left ventricular (LV) cardiomyocytes with fibrous and adipose tissue.

**Case summary:**

A 55-year-old male patient presented with recurrent chest pain and palpitations characterized by episodes of monomorphic ventricular tachycardia and T-wave inversion. Coronary angiography was conducted to rule out myocardial ischaemia as the cause of chest pain. Echocardiography results revealed ventricular aneurysm formation at the apex of the left ventricle. Structural alterations of the cardiac magnetic resonance were consistent with the diagnosis of arrhythmogenic left ventricular cardiomyopathy with LV alterations without right ventricular involvement. Pathological staining of the lesion area further confirmed the diagnosis of IALVC. The TTN1 c.17617 C>A mutation in arrhythmogenic cardiomyopathy was identified using whole exome sequencing. His symptoms improved by the treatments including implantable cardioverter defibrillator (ICD) implantation, radiofrequency ablation, and ventricular aneurysm resection.

**Discussion:**

The patient presented with IALVC with apical fibrofatty displacement and underwent surgical management, highlighting the importance of multimodal imaging, gene analysis, and histopathological findings for timely diagnosis, and emphasizing the benefits of life-saving therapy, including ICD implantation, radiofrequency ablation, and ventricular aneurysm resection. These findings contribute to a deeper understanding of the clinical presentation and outcome of IALVC.

**Learning points**

- Isolated arrhythmogenic left ventricular cardiomyopathy is a rare hereditary cardiomyopathy, which is difficult to diagnose and manage, and needs to be differentiated from complications of myocardial infarction.
- The combination of multimodal imaging, including echocardiography, cardiac magnetic resonance imaging, and timely capture of ventricular tachycardia is critical for timely diagnosis.
- Individualized prognostication and risk stratification should be performed, and we emphasized that the combination of drug therapy, implantable cardioverter defibrillator implantation, cardiac ablation, and necessary surgical therapy could significantly improve the prognosis.

## Introduction

Arrhythmogenic cardiomyopathy (ACM) is a hereditary cardiomyopathy that is characterized by the replacement of ventricular cardiomyocytes with fibrous and adipose tissue, presentation of ventricular arrhythmias, and increased risk of sudden cardiac death. Arrhythmogenic cardiomyopathy is closely associated with mutations in desmosomal proteins, which lead to the detachment of cardiomyocytes and alteration of intracellular signal transduction.^1^ Initially, ACM was described as a right ventricular (RV) disease, also known as arrhythmogenic right ventricular cardiomyopathy (ARVC). Recently, left ventricular (LV) involvement has also been reported in 16–76% of ACM cases.^2^ It is estimated that the incidence of ACM is ∼1:2000–5000, and it is one of the major causes of sudden cardiac death in young individuals and athletes.^3^ Multimodality imaging, including electrocardiogram (ECG) and cardiac magnetic resonance imaging (CMRI), plays an important role in early diagnosis.^4^

The present report describes a patient with acute chest pain and palpitations who was initially misdiagnosed with acute myocardial infarction. He was then diagnosed with isolated arrhythmogenic left ventricular cardiomyopathy (IALVC). This report highlights the importance of early diagnosis and risk stratification of IALVC, as well as the need for personalized treatment.

## Summary figure

**Figure:**
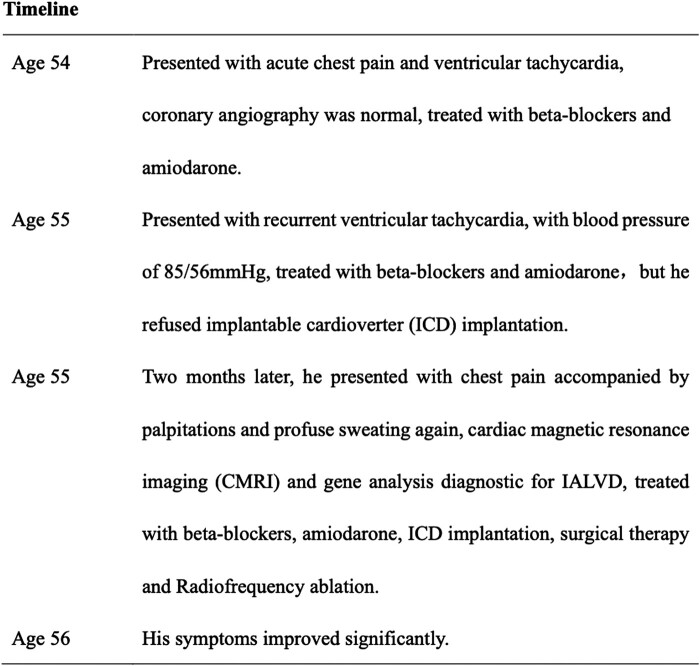


## Case presentation

A 54-year-old male patient presented with a history of recurrent palpitations and retrosternal pain accompanied by radiating pain, nausea, and vomiting. The heart rate at admission was 130 beats per minute (b.p.m.), and no other positive signs were found. The patient had no history of hypertension, diabetes, dyslipidaemia, or other cardiovascular diseases and denied any family history of transient syncope, coronary heart disease, malignant arrhythmia, or sudden death. The patient was admitted to a local hospital. The ECG results showed ventricular tachycardia (VT) at 213 b.p.m. Troponin level and myocardial enzyme profiles were normal. Coronary angiography showed normal coronary arteries (see Supplementary material online, *Figure S1*). Echocardiography showed thinning and protruding of the LV apex with local contradictory movements. Cardiac magnetic resonance imaging demonstrated abnormal systolic activity at the LV apex, while the structure and function of the right ventricle were normal. Non-coronary obstructive acute myocardial infarction was considered. The patient’s symptoms improved after undergoing treatment with metoprolol (47.5 mg/day), rosuvastatin (5 mg/day), and amiodarone (0.2 g/day). One year later, the patient experienced recurrent episodes of palpitations, with blood pressure of 85/56 mmHg. The patient denied having nausea, headache, syncope, and chest pain. Electrocardiogram results showed VT at 156 b.p.m. Paroxysmal ventricular tachycardia was then considered, and implantable cardioverter defibrillator (ICD) implantation was recommended. However, the patient declined the procedure. Instead, he was treated with metoprolol and amiodarone for cardioversion, which improved his symptoms.

**Figure 1:**
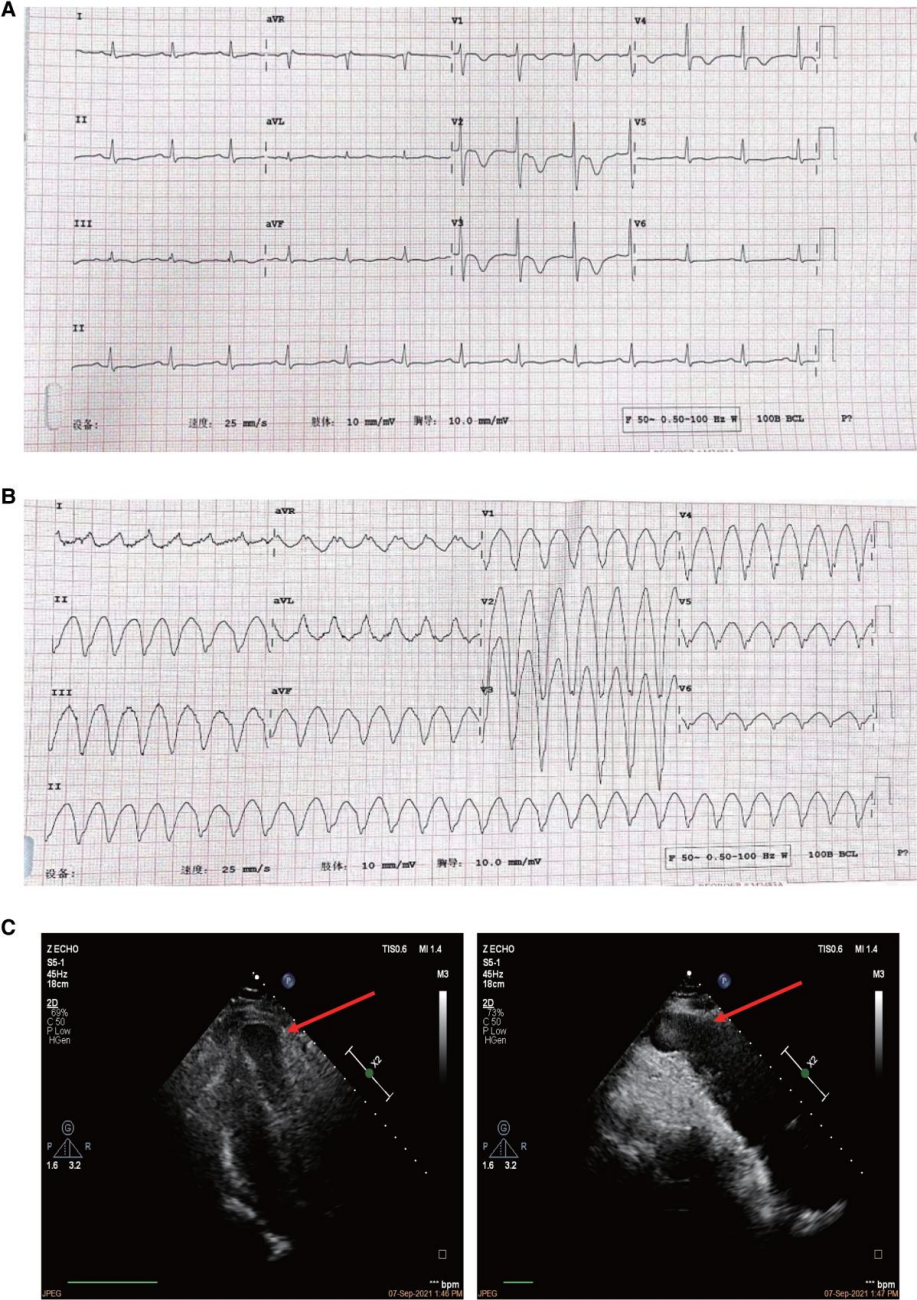
Patient’s electrocardiogram and echocardiogram characteristics at admission (2020.09). (*A*) Electrocardiogram findings recorded at the emergency department: sinus rhythm with heart rate of 80 b.p.m., inverted T-wave at V1–4, and depth of >1 mm. (*B*) Electrocardiogram results showed exercise-induced paroxysmal ventricular tachycardia. The VT is LBBB morphology with negative precordial concordance and wide QRS with notching, suggesting an inferoapical origin. (*C*) Echocardiography outcomes demonstrated left ventricular apical aneurysm and left atrial enlargement. The extent of ventricular aneurysm was ∼24 × 23 mm, and cardiac systolic function was not significantly affected.

**Figure 2:**
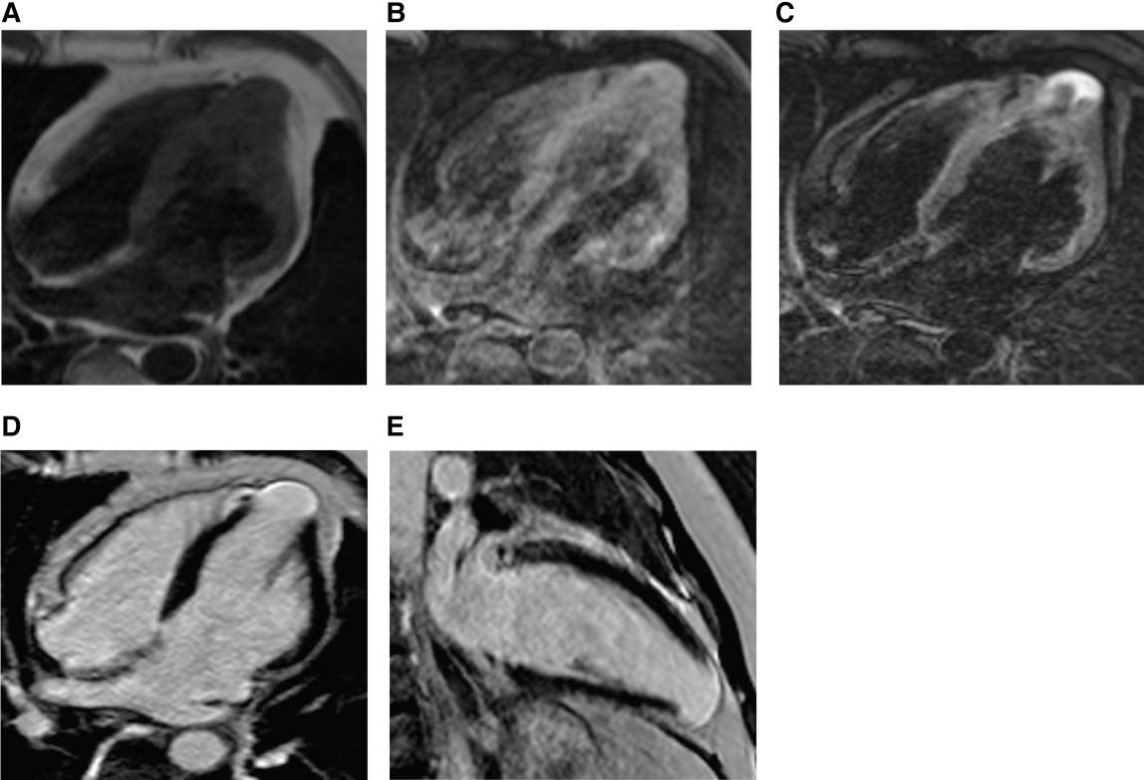
Cardiac magnetic resonance imaging (2020.09) revealed intramyocardial fatty spots in the left ventricle, especially at the apex. (*A* and *B*) T1-weighted black blood imaging with and without fat suppression. T1WI showed increased fat signal at the LV apex; (*C*) T2WI revealed a significant increase in apical signal with fat suppression. (*D* and *E*) Late gadolinium enhancement in LV apex.

**Figure 3:**
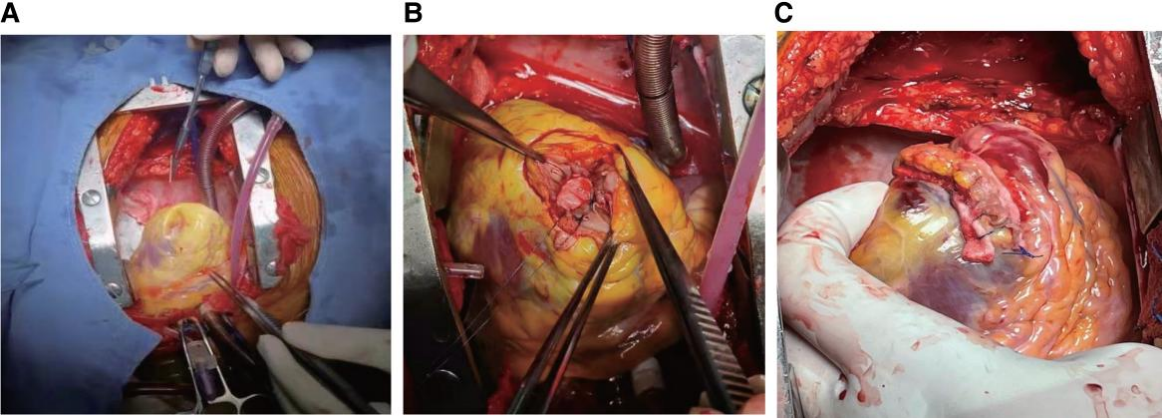
Apical ventricular aneurysm resection. (*A*) Thinning of apical ventricular aneurysm compared to its state before operation. (*B*) Surgical therapy for ventricular aneurysm. (*C*) Suturing after ventricular aneurysm resection.

After two months, the patient presented with chest pain, palpitations, and profuse sweating. A standard 12-lead ECG showed T-wave inversion in leads V1 to V4 with a depth exceeding 1 mm, with the deepest inversion in leads V1 and V2 at ∼5 mm (*Figure 1A*). During an episode of palpitations after admission, the ECG revealed paroxysmal ventricular tachycardia, which presented as left bundle branch block (LBBB) morphology with negative precordial concordance and wide QRS with notching, suggesting an inferoapical origin (*Figure 1B*). Echocardiography results demonstrated left atrial enlargement and LV apical aneurysm (*Figure 1C*). Cardiac magnetic resonance imaging showed LV apical dilatation, loss of systolic activity, and paradoxical motion during systole. Longitudinal relaxation time (T1) of the myocardium was then measured to indirectly determine the composition and pathological changes of myocardial tissue. Fat-suppressed T1-weighted images (T1WI) showed an increase in fat signal at the LV apex (*Figure 2A* and *B*). Additionally, fat-suppressed T2-weighted images (T2WI) demonstrated a significant increase in adipose signal and LV late gadolinium enhancement at the apex (*Figure 2C*–*E*). The patient’s myocardial enzyme spectrum and troponin level results were within normal limits. The troponin level was 0.007 ng/mL with normal range 0–0.03 ng/mL, NT-proBNP level was 85.5 pg/mL with normal range 0–100 pg/mL, creatine kinase-MB was 9 U/L with normal range 0–23 U/L, and creatine kinase-MM was 82 U/L, with normal range 0–174 U/L. Combined with previous medical history and investigation results, the IALVC diagnosis was strongly considered. Eventually, the patient accepted the treatment involving ICD, ventricular aneurysm resection, and radiofrequency ablation (*Figure 3*). The procedure revealed that the aneurysm was located on the anterior wall and apex of the left ventricle. Its size was ∼2.3 × 2.5 cm, and it exhibited paradoxical motion. Notably, no obvious myocardial infarction scar was observed. The aneurysm was completely excised, and the wall was found to be thin, with a neck dimension of ∼1.5 × 1 cm. A monopolar ablation pen was used for endocardial ablation near the neck of the LV aneurysm, bipolar radiofrequency ablation was utilized for LV aneurysm wall, and a 1.5 × 2 cm Bioren bovine pericardial patch was used for LV aneurysm incision closure.

**Figure 4:**
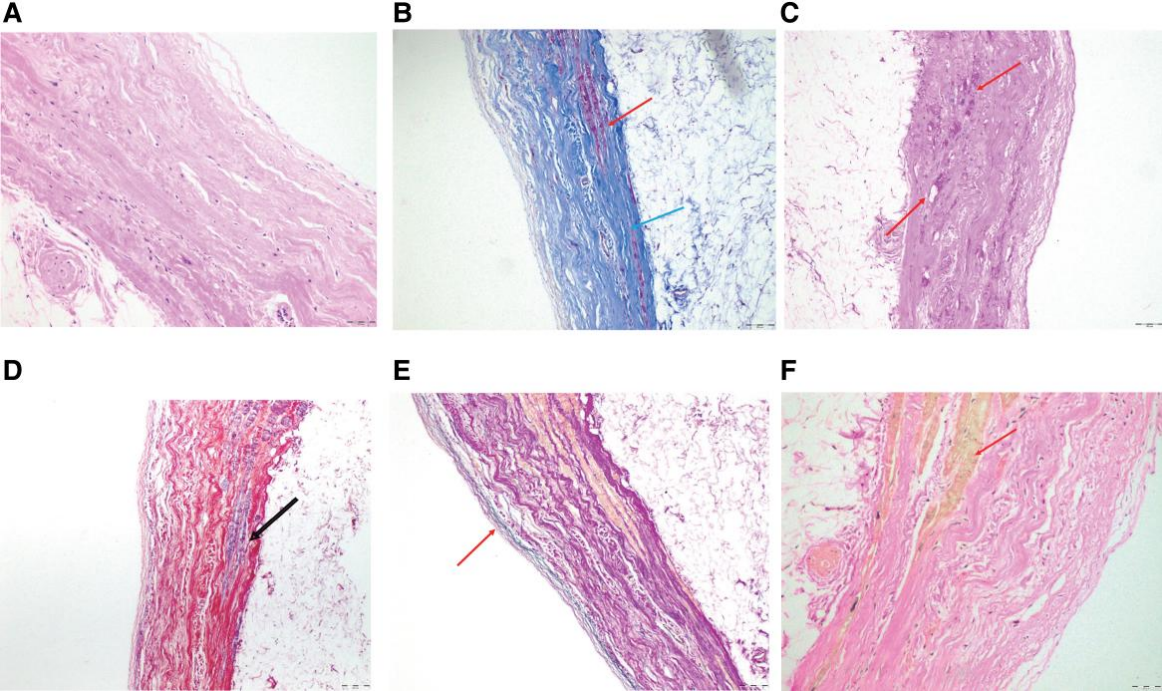
Pathological staining of ventricular aneurysm. (*A*, HE; *B*, Masson; *C*, PAS; *D*, PTAH; *E*, elastic staining; *F*, VG staining). HE staining revealed cardiomyocyte degeneration and rupture accompanied by proliferation of collagen fibres and limited infiltration of inflammatory cells. Masson and PAS staining further confirmed a significant reduction in cardiomyocyte numbers, which were largely replaced by collagen. Elastic staining showed endocardial preservation and loss of cardiomyocytes. VG staining indicated the presence of residual cardiomyocytes at the periphery of endocardium, with the majority being replaced by collagen fibres. Scale: 50 μm. HE, haematoxylin–eosin; PAS, periodic acid-Schiff; PTAH, phosphotungstic acid haematoxylin; VG staining, VanGieson staining.

**Figure 5:**
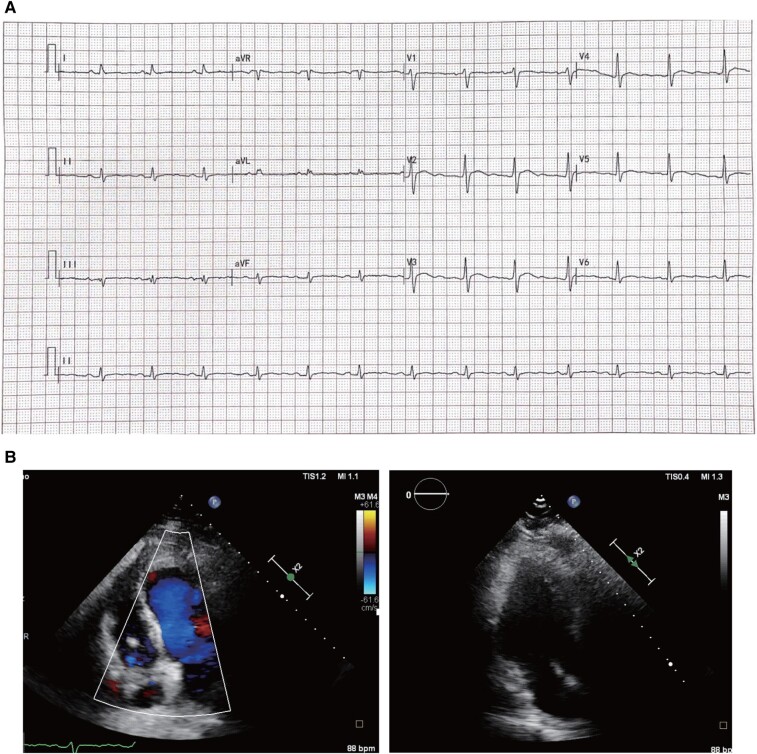
Electrocardiogram and echocardiography results after surgery. (*A*) Sinus rhythm and inverted T-wave return after treatment. (*B*) Echocardiography results showed a patch echo at LV apex.

Left ventricular aneurysm tissue was collected to conduct whole exome sequencing and histological staining. A novel mutation in TTN1 c.17617 C<A was identified, which resulted in the conversion from valine to phenylalanine at the 5873 site. The AlphaFold Protein Structure Database was used to predict the structure of TTN variants. The exon of this mutation site, as well as its upstream and downstream exons, was used to predict the domain structure. The results showed that the TTN1 mutation disrupted protein stability (see Supplementary material online, *Figure S2A*) and increased interaction between the mutation site (phenylalanine) and the remaining residues (see Supplementary material online, *Figure S2B* and *C*). As a result, this mutation site may be pathogenic and related to ACM. Histological staining showed a significant reduction and disappearance of cardiomyocytes in the LV aneurysm wall, which were predominantly replaced by collagen fibres and adipose tissue (*Figure 4*). Consequently, the patient was diagnosed with IALVC. The patient’s symptoms, including chest pain and palpitations, significantly improved after undergoing treatment mentioned above. After 6 months, the ECG results showed sinus rhythm, and echocardiography revealed postoperative patch echo and LV apical aneurysm disappeared (*Figure 5*).

## Discussion

Arrhythmogenic left ventricular cardiomyopathy (ALVC) is a condition characterized by myocardial dysfunction unrelated to myocardial ischaemia. The majority of isolated ALVC cases is located on the posterior basal wall (68%) and anterolateral wall of the left ventricle.^2^ Patients with IALVC frequently experience life-threatening arrhythmias, which are manifested as sustained monomorphic or polymorphic ventricular tachycardia with a left or right bundle branch block. Isolated arrhythmogenic left ventricular cardiomyopathy is strongly associated with a high incidence of sudden cardiac death, especially in young individuals and athletes.^5^ The pathological features of the lesion site are the loss of free wall cardiomyocytes and replacement by fibrotic and fatty tissue. The present case report provided a detailed account of the diagnosis and treatment of IALVC in a patient, emphasizing the importance of utilizing a combination of genetic analysis, essential histopathological findings, and multimodal imaging techniques, such as echocardiography and CMRI. Global longitudinal strain is the primary index of LV function in patients with ACM, which is assessed by speckle tracking analyses, is reported as the average peak negative systolic strain in 16 segments, and provides a more sensitive and accurate estimation of LV function than ejection fraction.^6^ When IALVC is suspected, as initial examination, we should focus on the echo of the lesion area. In addition, timely identification of VT is crucial in achieving an accurate diagnosis. Patients with acute chest pain and palpitations should be evaluated for ALVC if coronary angiography examination does not reveal ischaemic heart disease. Other conditions that can present without pouches in the apical structure, including pseudoaneurysm, Takotsubo cardiomyopathy, and ventricular aneurysm, also require further differential diagnosis. Post-myocardial infarction, congenital anomalies, pulmonary hypertension, and cardiac sarcoidosis should also be included in the differential diagnosis. Left ventricular aneurysm is one of the main manifestations of myocardial tissue loss in IALVC. Multimodality imaging, such as echocardiography, CMRI, and black blood imaging, is imperative in the timely assessment of ventricular aneurysm morphology, function, and tissue characterization.^7,8^ About 50% of patients with ACM had mutations in genes encoding cardiac desmosomal proteins, showing that gene variants, including DSP, RYR2, TTN, PKP2, DSC2, and DSG2, were closely related to ACM.^9,10^ DSP is the main component of desmosomes that interacts with PKP to link DSC and DSG to intermediate filaments.^11^ TTN is a candidate gene for ACM that encodes the titin protein, which attaches to the transition junction of the insertion disk. TTN mutations can lower Ig domain stability and lead to titin degradation, premature truncation, or frame shift changes at the C-terminus, ultimately leading to cardiomyocyte loss and replacement by fibrofatty tissue.^12^ Gene analysis is essential for further verifying the diagnosis of IALVC. Dilated cardiomyopathy and cardiomyopathy associated with neuromuscular diseases should be excluded due to the overlap in genetic background.^11^ Sarcoidosis and myocarditis can also cause life-threatening arrhythmias and should be excluded via genetic analysis.^11^

Individualized prediction and risk stratification should be performed in ARVC patients to identify those with a higher risk of sudden cardiac death or sustained ventricular tachycardia early. The potential predictors include age, gender, cardiac syncope in the prior six months, non-sustained ventricular tachycardia, a number of premature ventricular complexes in 24 h, and a number of ECG leads showing T-wave inversion.^13,14^ Current guidelines suggest that competitive or frequent high-intensity exercise should be limited in patients with ALVC.^15^ Drug management (including β-blockers and amiodarone) should be considered in case of palpitations, while ICD should be the first-line therapy, especially in patients with a history of cardiac arrest or sustained VT and those with severe RV or LV dysfunction.^16,17^ Ventricular aneurysm complications include cardiac rupture, which causes sudden death in ALVC patients. Ventricular aneurysm resection and catheter ablation might save lives in these patients.

## Conclusion

The present case provides a comprehensive description of the diagnosis and treatment of IALVC in a patient. It underscores the importance of using multimodal imaging, gene analysis, and histopathological findings for timely diagnosis of IALVC and emphasizes the critical advantages of drug interventions and other life-saving therapies, such as ICD implantation, radiofrequency ablation, and ventricular aneurysm resection. The findings contribute to a better understanding of the clinical presentation and outcome of ALVC, thereby facilitating more accurate and effective treatment.

## Lead author biography


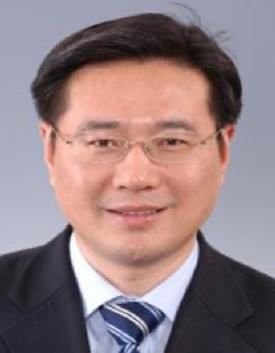
Prof. Yan Yan graduated from Shanghai Medical College of Fudan University, and completed his training under the direction of Prof. Junren Zhu. In 2005, he completed his post-doctoral training at Cleveland Heart Center in the USA. Currently, he is working in the Department of Cardiology, Zhongshan Hospital affiliated to Fudan University, and serves as a professor of Fudan University. He also served as the senior evaluation expert of Shanghai Science and Technology Commission, and presided over the National Natural Science Foundation of China and Shanghai Natural Science Foundation. He was the lead author of over 30 medical publications in leading journals including *Cell Research*.

## Supplementary material

Supplementary material is available at *European Heart Journal – Case Reports* online.

## Supplementary Material

ytad581_Supplementary_Data

## Data Availability

The data are available from the corresponding author on reasonable request.

## Notes

**Consent:** The authors confirmed that informed consent was obtained from the patient for the publication of this case report in accordance with the Committee on Publication Ethics (COPE) guideline.

**Funding:** This work was supported by Innovative Research Group Project of the National Natural Science Foundation of China (grant numbers: 81873538 and 82070463).
