# Supplementary material for: A case report of isolated arrhythmogenic left ventricular cardiomyopathy: phenotypes, diagnosis, and treatment
Source: Eur Heart J Case Rep. 2024 Feb 7;8(2):ytad581. doi: 10.1093/ehjcr/ytad581 (PMC10849070; doi:10.1093/ehjcr/ytad581)
Supplement: ytad581_Supplementary_Data [file ytad581_supplementary_data.zip › supplementary Figure 2.pdf]

A

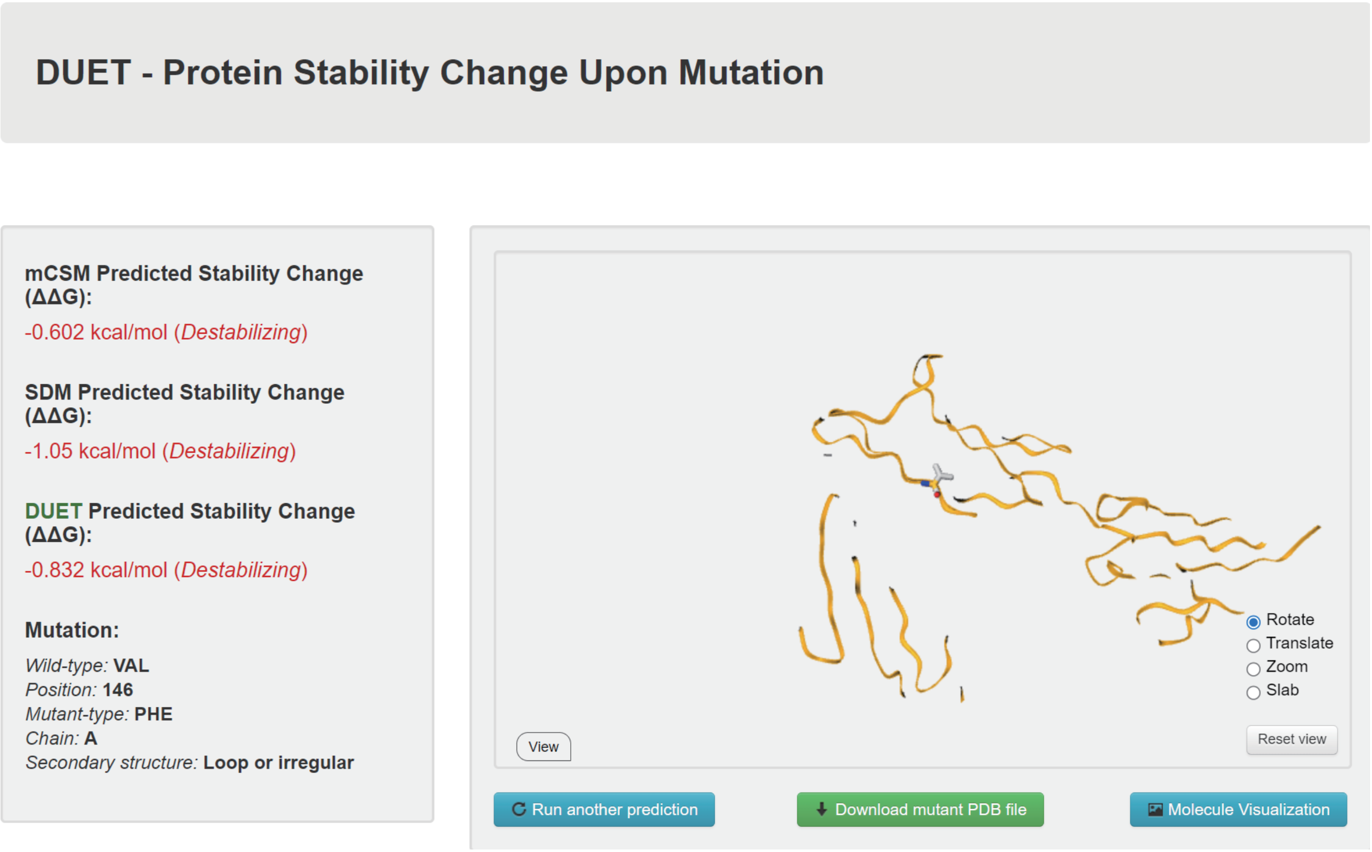
DUET - Protein Stability Change Upon Mutation
mCSM Predicted Stability Change (ΔΔG):
-0.602 kcal/mol (Destabilizing)
SDM Predicted Stability Change (ΔΔG):
-1.05 kcal/mol (Destabilizing)
DUET Predicted Stability Change (ΔΔG):
-0.832 kcal/mol (Destabilizing)
Mutation:
Wild-type: VAL
Position: 146
Mutant-type: PHE
Chain: A
Secondary structure: Loop or irregular
Rotate
Translate
Zoom
Slab
View
Reset view
Run another prediction
Download mutant PDB file
Molecule Visualization

B

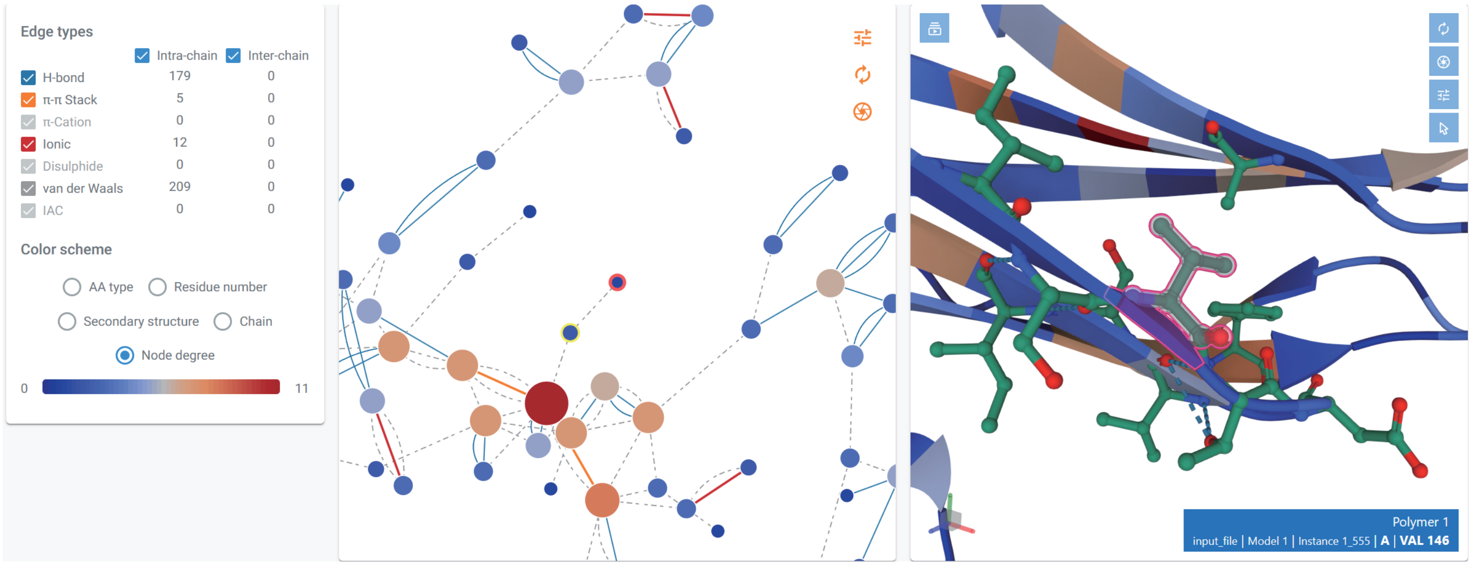
Edge types
Intra-chain
Inter-chain
H-bond 179 0
π-π Stack 5 0
π-Cation 0 0
Ionic 12 0
Disulphide 0 0
van der Waals 209 0
IAC 0 0
Color scheme
AA type
Residue number
Secondary structure
Chain
Node degree
0
11
Polymer 1
input_file | Model 1 | Instance 1_555 | A | VAL 146

C

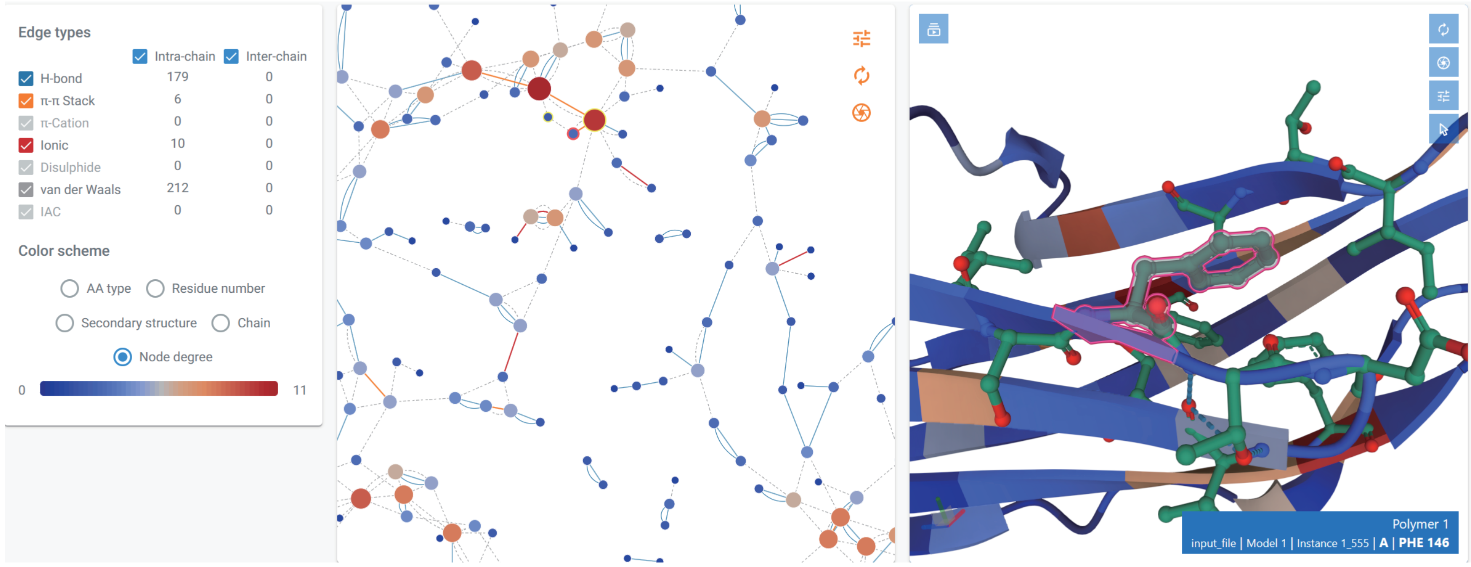
Edge types
Intra-chain
Inter-chain
H-bond 179 0
π-π Stack 6 0
π-Cation 0 0
Ionic 10 0
Disulphide 0 0
van der Waals 212 0
IAC 0 0
Color scheme
AA type
Residue number
Secondary structure
Chain
Node degree
0
11
Polymer 1
input_file | Model 1 | Instance 1_555 | A | PHE 146
