# Supplementary figures and images for: A case report of isolated arrhythmogenic left ventricular cardiomyopathy: phenotypes, diagnosis, and treatment
Source: Eur Heart J Case Rep. 2024 Feb 7;8(2):ytad581. doi: 10.1093/ehjcr/ytad581 (PMC10849070; doi:10.1093/ehjcr/ytad581)

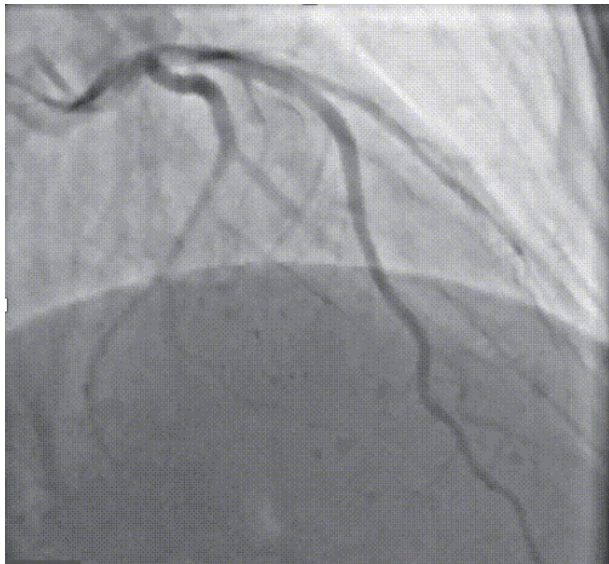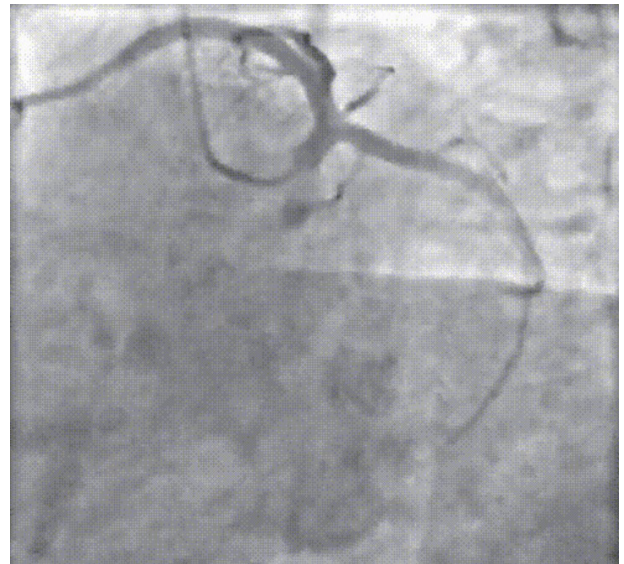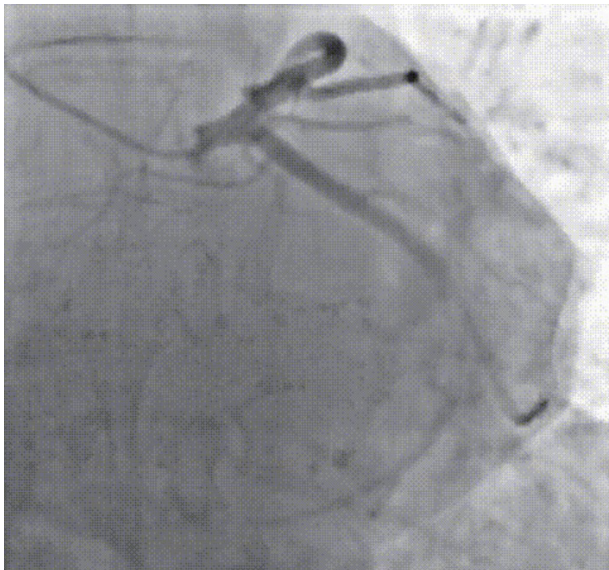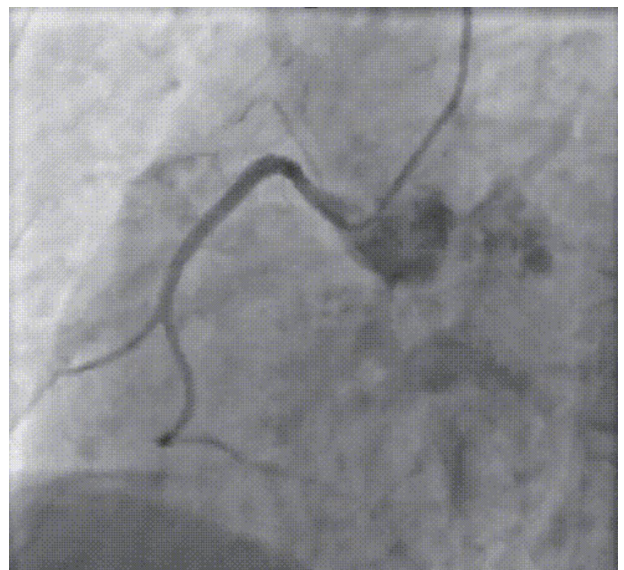

Supplement: ytad581_Supplementary_Data [file ytad581_supplementary_data.zip › renamed_bd063.pdf]
